# Genome sequences of pathogenic and non-pathogenic Pantoea ananatis strains in maize (Zea mays L.)

**DOI:** 10.1099/acmi.0.000709.v3

**Published:** 2025-02-14

**Authors:** Izabela Moura Duin, Viviane Yumi Baba, Katherine M. D'Amico-Willman, Fernanda Neves Paduan, Vanessa Hitomi Sugahara Rodrigues, Jose C. Huguet-Tapia, Jeffrey Bryant Jones, Marcelo G. Canteri, Rui Pereira Leite Júnior, Maria Isabel Balbi-Peña

**Affiliations:** 1 Entomology and Plant Pathology Department, North Carolina State University, Raleigh, NC, USA; 2 Programa de Pós-Graduação em Agronomia, Universidade Estadual de Londrina – UEL, Londrina, PR, Brazil; 3 Área de Proteção de Plantas, Instituto de Desenvolvimento Rural do Paraná/IAPAR-Emater, Londrina, PR, Brazil; 4 Plant Pathology Department, University of Florida, Gainesville, FL, USA; 5 Emerging Pathogens Institute, University of Florida, Gainesville, FL, USA

**Keywords:** bacterial leaf streak, bacterial pathogenesis, comparative genomics, maize white spot

## Abstract

We performed genome sequencing and comparative analysis of *Pantoea ananatis* strains isolated from corn leaves expressing typical bacterial leaf streak (BLS) and maize white spot (MWS) symptoms to confirm bacterial identity and to understand the relationship among these strains and *P. ananatis* strains isolated from different plant hosts in Brazil. In pathogenicity tests, strains 4.2 and 13.3 isolated from symptomatic BLS leaves were non-pathogenic on corn. In contrast, strain B13 isolated from MWS-diseased leaf tissue caused symptoms typical of MWS. Our comparative analysis revealed that all three strains are very genetically similar. The G+C (%) content of strains 4.2 and 13.3 was 53.5%, while the B13 content was 53.7%. Average nucleotide identity (ANI) analysis showed that strains B13 and 13.3, B13 and 4.2, and 4.2 and 13.3 shared ANIs of 99.17%, 99.15% and 99.99%, respectively. Strains 13.3, B13, and 4.2 shared ~99% ANI with *P. ananatis* type strain LMG 2665. To the best of our knowledge, these are the first genome sequences of *P. ananatis* strains isolated from corn in Brazil.

## Data summary

The genome sequence assemblies and raw read data have been deposited in the GenBank database under BioProject number PRJNA989117, and the accession numbers are JAUHJW000000000, JAUHJV000000000 and JAUHJU000000000 for *Pantoea ananatis* strains 13.3, 4.2 and B13 respectively.

## Introduction

The bacterial pathogen, *Pantoea ananatis*, is present in a large range of ecological niches and has been isolated from different environments, including water, soil, insects,and plants, as well as humans [1,2]. *P. ananatis* has a close association with plants as endo- or epiphytes, biocontrol agents, plant-growth promoters or true pathogens [3,6]. As a plant pathogen, *P. ananatis* causes economic losses on several agricultural crop plants, including mono- and dicotyledonous species [3].

In Brazil, Paccola-Meirelles *et al*. [7] first reported *P. ananatis* associated with maize white spot (MWS) in Brazil. More recently, bacterial leaf streak (BLS) caused by *Xanthomonas vasicola* pv. *vasculorum (Xvv*), another bacterial disease in corn, was reported in Brazilian corn-producing regions [8,9]. Interestingly, *P. ananatis* has been isolated from corn leaves infected with *Xvv,* but these strains do not cause disease on corn plants [10]. As stated in Ortiz-Castro *et al*. [11], studies are needed to explore the association *of P. ananatis* with corn, including the ability to cause disease, the interaction with other bacterial species and the potential as a biocontrol agent. The genetic mechanisms driving the interactions between *P. ananatis* and their hosts are poorly understood, as they can colonize and exploit several different environmental niches [1]. Previous comparative genomics studies were performed on *P. ananatis* strains to identify pathogenicity-related genes that could influence the ability of the bacteria to colonize and interact with the plant hosts [1,12, 13].

In this study, we performed whole-genome sequencing of Brazilian *P. ananatis* strains isolated from corn leaves symptomatic of BLS or MWS. These data were used to perform comparative genomic analyses with *P. ananatis* strains isolated from corn in Brazil with other *P. ananatis* strains to determine their phylogenetic relationships and identify strain-specific genes present in Brazilian strains pathogenic and non-pathogenic on corn. These are the first genome sequences of *P. ananatis* strains isolated from corn in Brazil.

## Methods

Strains 13.3 and 4.2 belong to the plant pathogenic bacterial collection at the Laboratório de Bacteriologia e Diagnose em Fitossanidade do Instituto de Desenvolvimento Rural do Paraná - IAPAR/Emater in Londrina, PR, Brazil. The strains were isolated from corn leaves symptomatic of BLS and collected in corn fields in the western region of Parana, Brazil [8,9], and identified and characterized as *P. ananatis* through genomic and phenotypic analyses presented in this study. The *P. ananatis* strain B13 was provided by the Coleção de Bactérias Fitopatogênicas do Instituto de Ciências Agrárias da Universidade Federal de Uberlândia in Uberlância, MG, Brazil. Strain B13 was isolated from corn leaves symptomatic of MWS and collected in Goiás State, Brazil, in 2010. Silva and Tebaldi [14] identified and characterized B13 based on cultural, biochemical and molecular analysis and determined pathogenicity in corn.

To test pathogenicity of strains 13.3 and 4.2, a pathogenicity assay was performed using detached corn leaves *cv*. ‘Formula’ in a micro-humidity chamber [15], including strain B13 as a positive control. The strains were grown in tryptic soy broth (TSB) medium overnight at 30 °C with shaking at 60 r.p.m., after which 1 ml of the pre-inoculum was transferred to 100 ml of TSB medium and incubated under the same conditions for 4 h. A saline solution (NaCl 0.85%) was then added to the bacterial culture [1 : 1 (v/v)]. Corn leaves were collected 35 days after seedling emergence and were placed on top of a piece of filter paper and then kept between two pieces of plexiglass, forming a micro-humidity chamber. The top piece of plexiglass contained 12 holes, each 0.3 cm in diameter, through which a needle was used to wound the surface of the leaf. Following this, a 20 µl aliquot of bacterial inoculum (10^8^ c.f.u. ml^−1^) was pipetted into the well onto the wound site. TSB medium/saline solution was used as a negative control. The leaves were kept in the plexiglass chambers for 72 h at 25 and 15 °C, alternating every 12 h, and were examined daily for disease symptom development.

For total DNA extraction, strains 13.3, 4.2 and B13 were initially grown on NA medium for 48 h at 28 °C. An overnight culture was prepared from the plate in nutrient broth and incubated at 28 °C for 16 h with shaking at 200 r.p.m. A 1 ml aliquot of the overnight culture was pelleted, and total DNA was extracted from the pellet using the DNeasy Blood and Tissue Kit (Qiagen, Hilden, Germany) following the manufacturer’s instructions. DNA samples from strains 13.3 and 4.2 were shipped to The Microbial Genome Sequencing Center (Pittsburgh, PA, USA) for library preparation and sequencing on the MiSeq Illumina Sequencing NextSeq 2000 platform. DNA isolated from strain B13 was sequenced on the Illumina MiSeq platform at the Soil Biotechnology Laboratory of Embrapa Soja, Londrina, PR, Brazil.

Sequence quality was analysed using FastQC [16], and reads were trimmed using Trim Galore (v. 0.6.5) with default parameters (Krueger F. Trim-Galore, accessible at http://www.bioinformatics.babraham.ac.uk/projects/trim_galore/). Trimmed reads were *de novo* assembled using SPAdes v. 3.15.5 incorporated in the Uniclycler pipeline [17] with default parameters. Genome quality and G+C (%) content were assessed using quast version 5.3 [18]. To confirm the taxonomic classification of these strains as *P. ananatis*, GTDB-tk v. 2.4.0 was used on the Galaxy platform 2024 [19,21].

Publicly available whole-genome sequences for additional *P. ananatis* strains were obtained from NCBI GenBank (Table 1). Strain PA13, isolated from *Oryza sativa* in Korea [22], is the reference strain for this species and is classified as pathogenic in rice. Strain LMG 2665 is the type strain isolated from pineapple (NCBI RefSeq assembly GCA_000661975.1).

**Table 1.** List of *Pantoea ananatis* strains included in this study

Strain	Host	Reaction on original host	Origin	GenBank accession	Reference
B13	*Zea mays*	Pathogenic	Brazil	NZ_JAUHJU000000000.1	This study
4.2	*Zea mays*	Non-pathogenic	Brazil	NZ_JAUHJV000000000.1	This study
13.3	*Zea mays*	Non-pathogenic	Brazil	NZ_JAUHJW000000000.1	This study
FDAARGOS 680	Not informed	Not informed	USA	ch: NZ_CP054912.1pl: NZ_CP054909.1pl: NZ_CP054910.1pl: NZ_CP054911.1	Kerrigan *et al*. (Unpublished)
S6	*Zea mays*	Growth-promoter	Austria	ch: NZ_CVNF01000021.1	Sheibani-Tezerji *et al*. [13]
S7	*Zea mays*	Pathogenic	Austria	ch: NZ_CVNG01000003.1	Sheibani-Tezerji *et al*.[13]
S8	*Zea mays*	Commensal	Austria	ch: NZ_CVNH01000026.1	Sheibani-Tezerji *et al*.[13]
LCFJ-001	*Morus alba*	Not informed	China	ch: NZ_CP066803.1	Liu and Luo (Unpublished)
LMG 20103	*Eucalyptus* sp.	Pathogenic	South Africa	ch: NC_013956.2	De Maayer *et al*. [1]
OC5a	*Allium cepa*	Pathogenic	USA	ch: NZ_CP059084.1pl: NZ_CP059083.1pl: NZ_CP059085.1pl: NZ_CP059086.1	Asselin *et al*. (Unpublished)
PA13	*Oryza sativa*	Pathogenic	Korea	ch: NC_017554.1	Choi *et al*. [22]
				pl: NC_017553.1	
PNA 97–1R	*Allium cepa*	Pathogenic	USA	ch: NZ_CP020943.2pl: NZ_CP020944.2pl: NZ_CP020945.2	Stice *et al*. [31]
PNA 99–7	*Allium cepa*	Non-pathogenic	USA	ch: NZ_NMZW01000001.1	Stice *et al*. [31]
LMG 2665^T^	*Ananas comosus*	Not informed	Philippines	ch: JMJJ01000017.1	De Maayer *et al*. [1]

To examine variability among genomic regions in strains 13.3, 4.2 and B13, a whole-genome alignment was performed using mauve v. 20150226 with default parameters and the reference strain, PA13. To determine the average nucleotide identity (ANI) among strains 13.3, 4.2 and B13 and the ten additional *P. ananatis* strains, pyani v. 0.2.12 was used with the *-m ANIb* option [23]. The resulting matrix of pairwise identities was visualized using pheatmap v. 1.0.12 in R v. 4.4.1 [24]. Genome annotations were performed using prokka v. 1.14.6 with default parameters to generate gff files for each strain [25]. A pangenome was constructed for the 13 strains using panaroo v. 1.5.0 with *--clean-mode* set to *strict* [26]. The core genome alignment was then used to infer a maximum likelihood phylogeny using iqtree v. 2.3.4 first to determine the best-fitting nucleotide substitution model based on Bayesian information criterion scores and then repeated using the selected model with bootstrapping and the approximate likelihood ratio test set to 1000 [27].

## Results

**Fig. 1.**
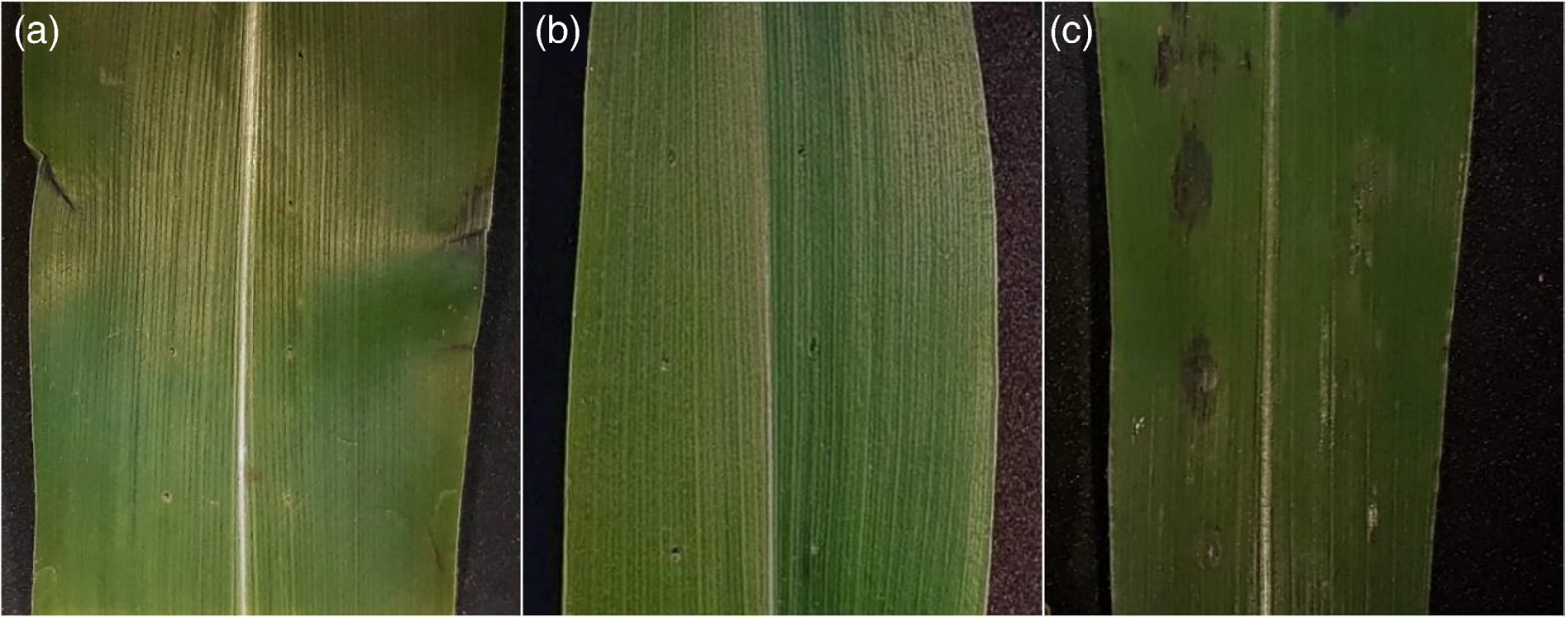
Pathogenicity assay inoculating *Pantoea ananatis* strains: (**a**) 13.3, (**b**) 4.2 and (**c**) B13 onto detached corn leaves *cv*. ‘Formula’. Images show symptom development 72 h post-inoculation.

The pathogenicity assay showed that inoculation with *P. ananatis* strains 13.3 and 4.2 did not cause any symptoms of either MWS or BLS (Fig. 1a, b). However, strain B13 causes MWS symptoms (water-soaked lesions) when inoculated on corn leaves (Fig. 1c). Inoculation with TSB medium/saline solution did not show symptom development.

Following genome sequencing and assembly, whole genomes were produced for strains 13.3, 4.2 and B13. The genome sizes ranged from 4.5 to 5 Mb, with coverage between 108× and 346× (Table 2). The quast quality analysis revealed that the genomes are 99.34%–100% complete with N50 >232 000 bp (Table 2). The prokka annotation identified 5004, 5133 and 4579 CDSs, in strains 13.3, 4.2 and B13, respectively (Table 2). The genome sequences for the three *P. ananatis* strains sequenced in this study were deposited in the GenBank database under accession numbers JAUHJV000000000, JAUHJW000000000 and JAUHJU000000000 (Table 2). Whole-genome alignment in mauve produced 66 locally colinear blocks among the four genomes with a minimum weight of 48 (Fig. S1, available in the online Supplementary Material).

**Table 2.** Genome assembly statistics for three *Pantoea ananatis* strains isolated from corn in Brazil

Attribute	*Pantoea ananatis* strain		
	**13.3**	**4.2**	**B13**
Total length (bp)	4 925 456	5 022 883	4 580 233
Sequence coverage	142	108	346
Number of contigs	23	46	34
G+C (%)	53.5	53.5	53.7
N50	334 641	340 846	232 217
L50	5	4	8
Largest contig	965 723	892 727	550 347
Completeness (%)	99.34	100	99.88
CDS	5 004	5 133	4 579
RNAs	67	71	73
GenBank accession no.	JAUHJW000000000	JAUHJV000000000	JAUHJU000000000

**Fig. 2.**
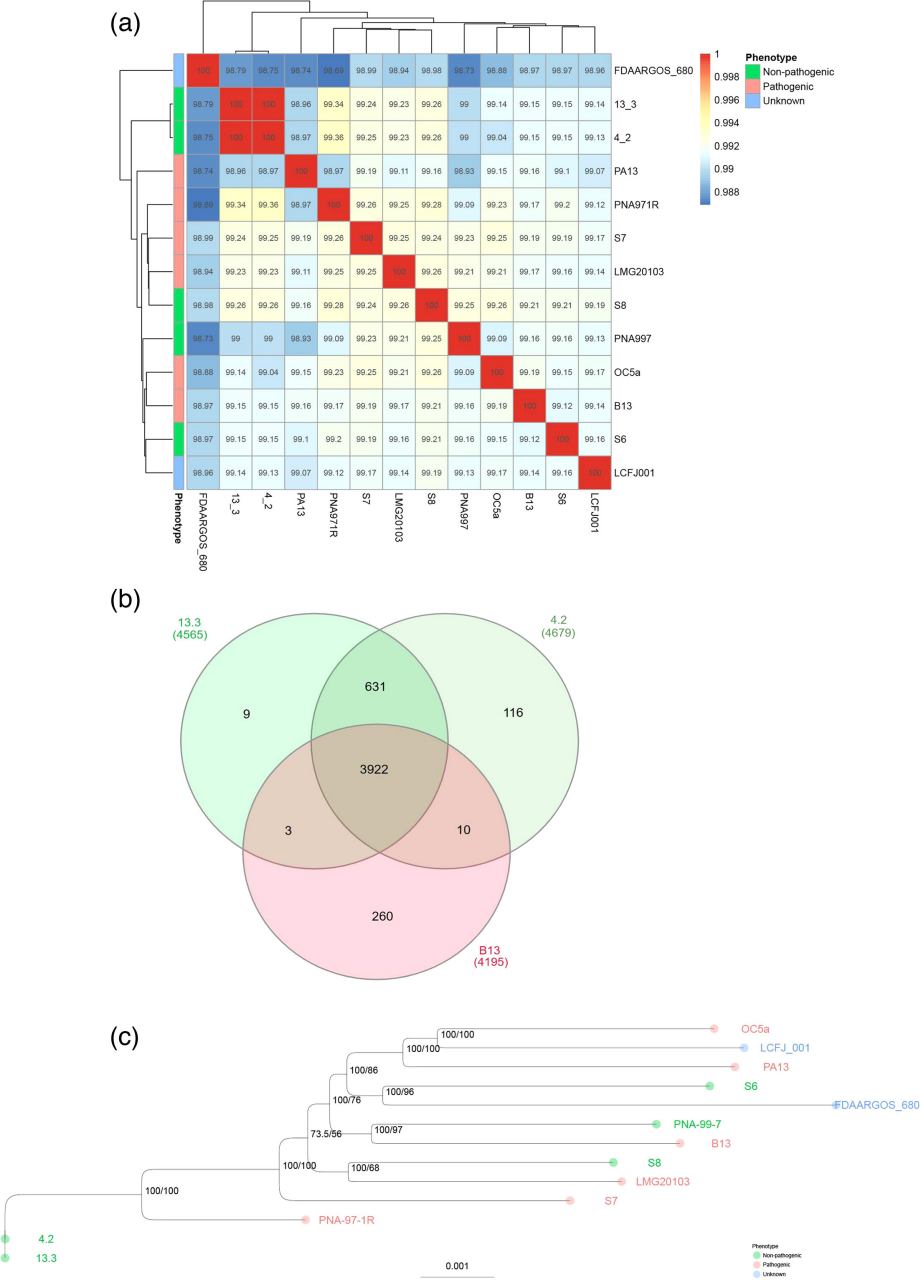
(**a**) Heatmap showing the percent ANI values calculated using pyani for 13 *Pantoea ananatis* strains. (**b**) Venn diagram showing the shared orthologous genes in the pangenome of the *P. ananatis strains* B13, 4.2 and 13.3. (**c**) Maximum likelihood phylogenetic tree inferred for 13 *P. ananatis* strains using the core genome alignment. Bootstrap and aLRT values are shown at the nodes of the tree. Tip labels are coloured based on pathogenicity phenotype, where green labels represent non-pathogenic strains, red labels represent pathogenic strains and green labels represent strains with unknown phenotypes.

The results of the ANI analysis showed that *P. ananatis* strains B13 and 13.3, B13 and 4.2, and 4.2 and 13.3 shared nucleotide identities of 99.17%, 99.15% and 99.99%, respectively. The ANIs of the three Brazilian strains (13.3, 4.2 and B13) compared with the other *P. ananatis* strains, including reference strain PA13 and type strain LMG 2665, were >98%, confirming that the strains all fall within the same species using a 95% species cutoff (Fig. 2a). Results of the GTDB-tk analysis further confirm the taxonomic classification of strains 4.2 and 13.3 with an assigned classification of *P. ananatis*.

Using the gene_presence_absence output file from panaroo [26], the number of total genes in the pangenome was determined, and the number of genes present or absent in the pathogenic strain (B13) compared with the non-pathogenic strains (4.2 and 13.3) was identified (Table S1). A Venn diagram was created using the web-based tool InteractiVenn [28], showing the number of genes shared among *P. ananatis* strains B13, 13.3 and 4.2 (Fig. 2b). *P. ananatis* strains B13, 4.2 and 13.3 shared 3922 orthologous genes and had 260, 116 and 9 unique genes, respectively (Fig. 2b).

A maximum likelihood tree was inferred using a multiple sequence alignment of the core genome of 13 *P*. *ananatis* strains, including B13, 13.3 and 4.2, to estimate the evolutionary relationships between these strains. Interestingly, the results of this analysis do not show clustering based on the pathogenicity of the strains using the currently available *P. ananatis* genomes and available pathogenicity data (Fig. 2c). Strain B13 clustered most closely with non-pathogenic strain PNA-99–7 isolated in the USA on *Allium cepa* (onion) (Fig. 2c). Strains 4.2 and 13.3 cluster most closely with each other and then with pathogenic strain PNA-97–1R, which was also isolated in the USA on *A. cepa* (Fig. 2c).

## Discussion

*P. ananatis* strains vary in their interactions with plant hosts, ranging from true pathogenic interactions to mutualistic associations [1]. In our study, we performed genome sequencing and comparative analysis of three Brazilian *P. ananatis* strains isolated from corn leaves expressing typical symptoms of BLS, caused by *X. vasicola* pv. *vasculorum* (*Xvv*), and MWS, caused by *P. ananatis*. This analysis confirmed the identity of the bacterial strains and contributed to our understanding of the relationships between pathogenic and non-pathogenic *P. ananatis* strains. Lang *et al*. [10] reported that *P. ananatis* has frequently been isolated from corn leaves infected with *Xvv*. The interaction between these two bacterial species is still unclear. Ortiz-Castro . [29] observed that co-infiltration of *Xvv* and *P. ananatis* resulted in a significant decrease in *Xvv* aggressivity, and inoculations with *P. ananatis* alone under the same conditions resulted in no symptom development. These results suggested that *P. ananatis* might exhibit antagonistic activity against *Xvv* while competing for space or nutrients. Additional studies are needed to better understand this antagonistic relationship between *P. ananatis* and *Xvv* and the impact on disease development in corn [10,11].

Although the three *P. ananatis* strains tested in this study were originally isolated from diseased corn leaves, they showed distinct phenotypes when inoculated onto corn leaves to test for pathogenicity. While strains 4.2 and 13.3 did not show typical symptoms of MWS after inoculation, strain B13 induced symptoms typical of the disease. Lang *et al*. [10] observed similar results on corn plants inoculated with *P. ananatis* strains isolated from corn leaves symptomatic of BLS. In a 2015 study, Sheibani-Tezerji *et al*. found that endophytic *P. ananatis* strains from maize seeds were highly genetically similar but showed distinct phenotypes in their interactions with host plants. The basis for the variation in pathogenicity of *P. ananatis* strains remains unclear; however, recent work suggests that the complex nature of the plant–bacteria interaction in this system could be related to the physiological status of the plant and other biotic and abiotic factors such as phyllosphere microbial community structure and environmental conditions [30].

Our comparative analysis revealed that all *P. ananatis* strains analysed in this study shared >98.5% similarity based on ANI values. The three strains (4.2, 13.3 and B13) isolated from corn in Brazil shared >99% similarity based on ANI analysis. Interestingly, strains 4.2 and 13.3 shared the highest similarity first with each other and then with reference strain PA13, a pathogenic strain isolated from rice in Korea, while strain B13 was most similar to strain PNA971R, a pathogenic strain isolated from onion in the USA. The pangenome analysis showed 3922 core genes shared between strains B13, 13.3 and 4.2, which represents ~80% of the pangenome. This suggests high similarity within the pangenome among the three strains [1,13]. Nonetheless, *P. ananatis* strains 13.3 and 4.2 shared a higher number of genes (631) within the pangenome compared with B13, suggesting that their genomes are highly conserved. Conversely, B13 shared only three and ten genes with strains 13.3 and 4.2, respectively.

We compared the genomes of three *P. ananatis* strains, isolated from corn leaves in Brazil, that show distinct interactions with the host plant despite their high genetic similarity. Additional studies are needed to better understand the niche adaptation and the interaction between *P. ananatis* and *Xvv* and the genes underlying the pathogenicity of strain B13.

## supplementary material

10.1099/acmi.0.000709.v3 Uncited Fig. S1.

10.1099/acmi.0.000709.v3 Uncited Table S1.

## Abbreviations

ANI: average nucleotide identity
BLS: bacterial leaf streak
MWS: maize white spot
TSB: tryptic soy broth
