# Supplementary figures and images for: Genome sequences of pathogenic and non-pathogenic Pantoea ananatis strains in maize (Zea mays L.)
Source: Access Microbiol. 2025 Feb 14;7(2):000709.v3. doi: 10.1099/acmi.0.000709.v3 (PMC11829076; doi:10.1099/acmi.0.000709.v3)

Supplementary Figure 1. Whole genome alignment for four *Pantoea ananatis* strains in mauve.

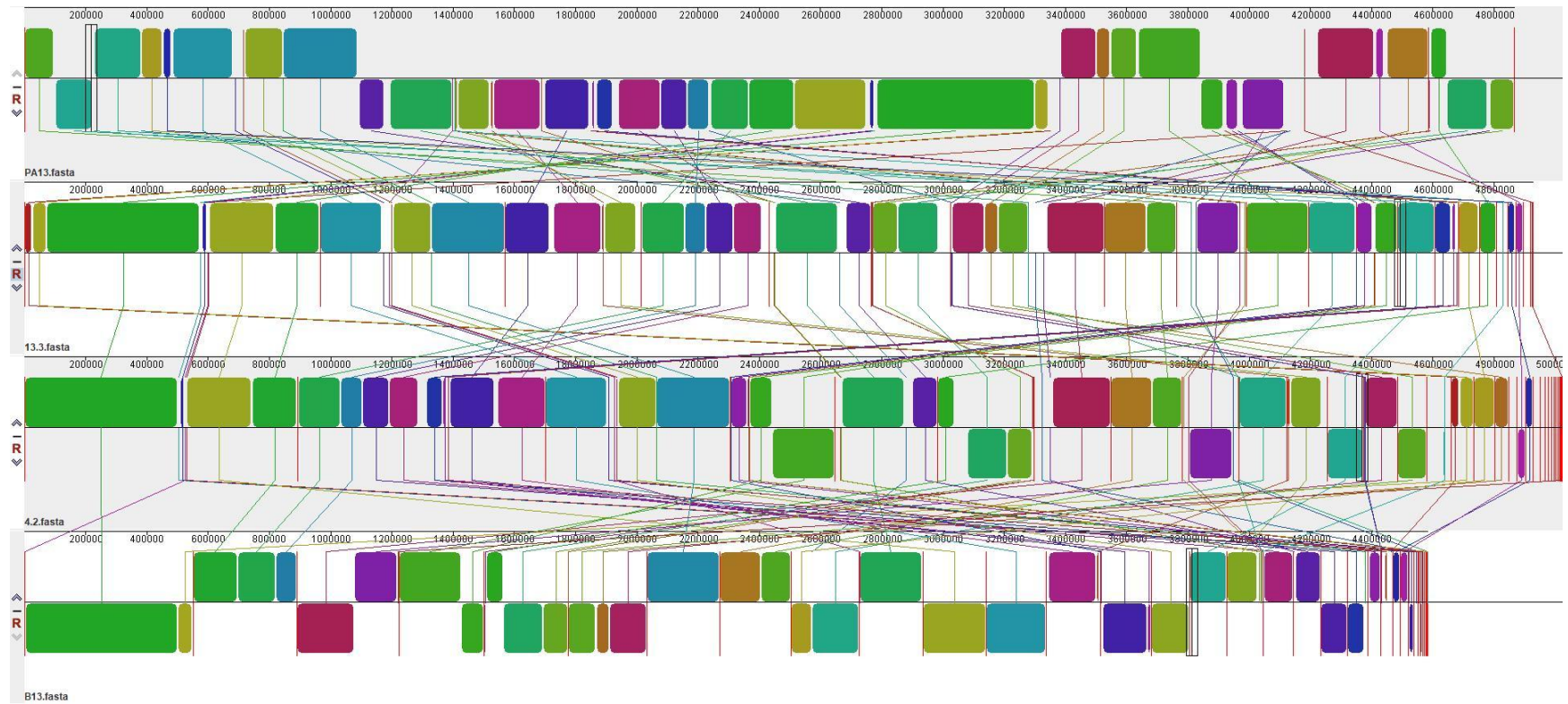

Supplement: Uncited Fig. S1. [file acmi-7-00709-s001.pdf]
